# Genetic networks in Parkinson’s and Alzheimer’s disease

**DOI:** 10.18632/aging.102943

**Published:** 2020-03-23

**Authors:** Jack Kelly, Rana Moyeed, Camille Carroll, Shouqing Luo, Xinzhong Li

**Affiliations:** 1 Faculty of Health: Medicine, Dentistry and Human Sciences, Plymouth University, Plymouth PL6 8BU, UK; 2 Faculty of Science and Engineering, Plymouth University, Plymouth PL6 8BU, UK; 3 School of Science, Engineering and Design, Teesside University, Middlesbrough TS1 3BX, UK

**Keywords:** network analysis, Parkinson’s disease, Alzheimer’s disease, gene co-expression, transcriptome analysis

## Abstract

Parkinson’s disease (PD) and Alzheimer’s disease (AD) are the most common neurodegenerative diseases and there is increasing evidence that they share common physiological and pathological links. Here we have conducted the largest network analysis of PD and AD based on their gene expressions in blood to date. We identified modules that were not preserved between disease and healthy control (HC) networks, and important hub genes and transcription factors (TFs) in these modules. We highlighted that the PD module not preserved in HCs was associated with insulin resistance, and *HDAC6* was identified as a hub gene in this module which may have the role of influencing tau phosphorylation and autophagic flux in neurodegenerative disease. The AD module associated with regulation of lipolysis in adipocytes and neuroactive ligand-receptor interaction was not preserved in healthy and mild cognitive impairment networks and the key hubs *TRPC5* and *BRAP* identified as potential targets for therapeutic treatments of AD. Our study demonstrated that PD and AD share common disrupted genetics and identified novel pathways, hub genes and TFs that may be new areas for mechanistic study and important targets in both diseases.

## INTRODUCTION

Alzheimer’s disease (AD) is the most common neurodegenerative disease (ND) and dementia, accounting for 60-80% of dementia cases. AD is characterized pathologically by accumulation of extracellular amyloid-β1 (Aβ) and deposits of intracellular tau neurofibrillary tangles [1]. In the US, the number of people living with AD is projected to increase from 5.5 million in 2018 to 13.8 million by 2050 [2]. Gradual progressive memory loss is the most common clinical symptom of AD, which eventually affects other cognitive functions such as communication and movement. There are currently many promising advances in the understanding of AD, including discovery of novel biomarkers [3, 4] and analysis of underlying biological mechanisms [5].

Parkinson’s disease (PD) is the second most prevalent ND affecting approximately 145,000 people in the UK [6], and PD patient numbers are predicted to increase by 87.6% between 2015 and 2065 [6]. In the US, the number of PD cases are predicted to increase from 680,000 to 1,238,000 by 2030 [7]. The accumulation of α-synuclein in neurons in the form of Lewy bodies is the main neuropathologic hallmark of PD [8]. Primarily, PD affects the motor systems of the central nervous system (CNS) as a result of the death of dopamine generating cells in the midbrain substantia nigra (SN) [8].

There is growing evidence that AD and PD share many common characteristics [9]. Around 80% of PD patients will develop dementia, with an average time of onset 10 years from PD diagnosis [10]. We have recently shown that PD and AD share significant common differentially expressed genes (DEGs), disturbed pathways including the sirtuin signaling pathway, and identified *REST* as an important upstream regulator in both diseases [11]. AD and PD are both age-related diseases that have hallmarks of protein aggregation. In fact over 60% of AD cases are accompanied by the formation of Lewy bodies and α-synuclein is found as a non-amyloid component within AD amyloid plaques [12]. In addition, there are certain genetic variants that increase the risk of both AD and PD, for example the strong risk factor for AD, APOE4, has been shown to be related to cognitive decline in PD [13].

Gene co-expression relationships contain a wealth of information that univariate methods like differential expression analysis cannot detect [14]. Weighted gene co-expression network analysis (WGCNA) is a popular tool used in systems biology to construct co-expression gene networks which can detect gene modules as well as identify key genes and hubs within these modules [15]. WGCNA has been used to find strong evidence for mitochondrial dysfunction and chronic low grade innate immune response in AD [16]. In addition, Chatterjee et al. [17] identified 11 hub genes by using WGCNA in frontal cortex and SN brain samples of PD patients.

**Figure 1:**
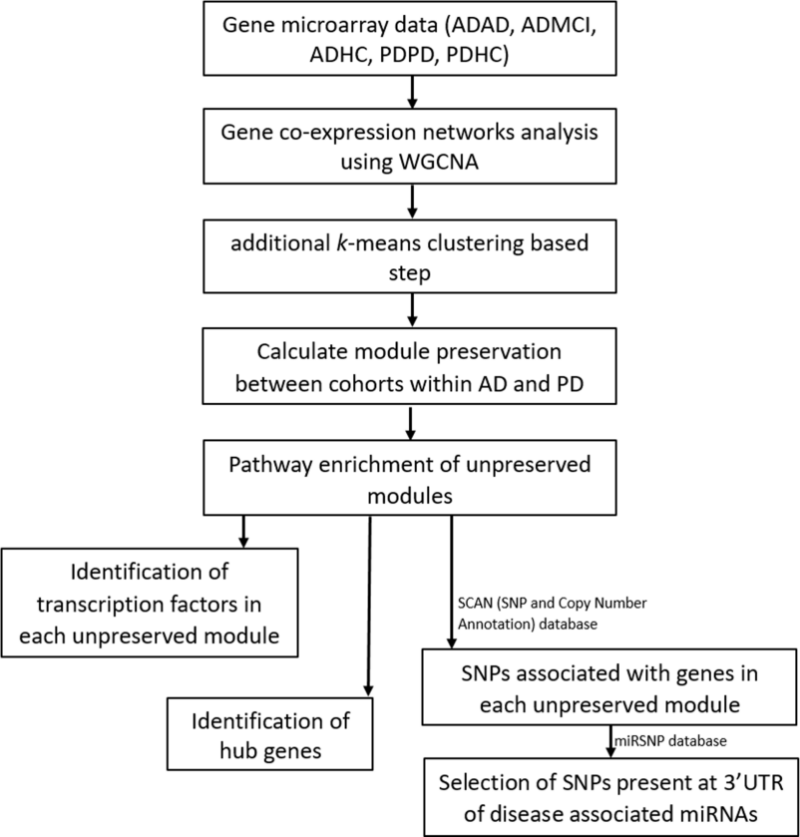
**Workflow of our analysis.** Filtered and normalized microarray data were separated into five datasets: AD disease (ADAD), healthy control (ADHC) and MCI (ADMCI) data from the AD dataset, and the PD disease (PDPD) and healthy control (PDHC) data from the PD dataset. On each dataset gene co-expression networks analysis was performed using the WGCNA R package [15]. An additional *k*-means correction step to reduce number of misplaced genes [70] was then performed and module preservation between cohorts within AD and PD was found using NetRep (v.1.2.1) [18]. The pathways associated with non-preserved modules were then found using the Enrichr web tool [19, 20] and hub genes and transcription factors in these non-preserved modules identified. The SCAN (single nucleotide polymorphism (SNP) and Copy number ANnotation) database) database [25] was used to find SNPs associated with the genes in each non-preserved module and these SNPs used to search the MiRSNP database to find the SNPs at 3’ UTR of disease associated miRNAs.

To date there have been no studies investigating PD and AD using gene expression network simultaneously to reveal potential shared biological process and pathology. In this study we analyzed gene co-expression networks based on PD and AD blood microarray data and identified common genetic networks between both diseases. See our analysis workflow illustrated in Figure 1. Compared to brain tissues, blood tissue is easier to access from patients with ND, and publicly available AD and PD blood datasets have a large enough sample size to construct reliable and robust networks. Our network analysis expands on standard WGCNA and hub detection approach which can robustly find key processes and genes that are associated with both PD and AD.

## RESULTS

### Gene co-expression network construction

**Figure 2:**
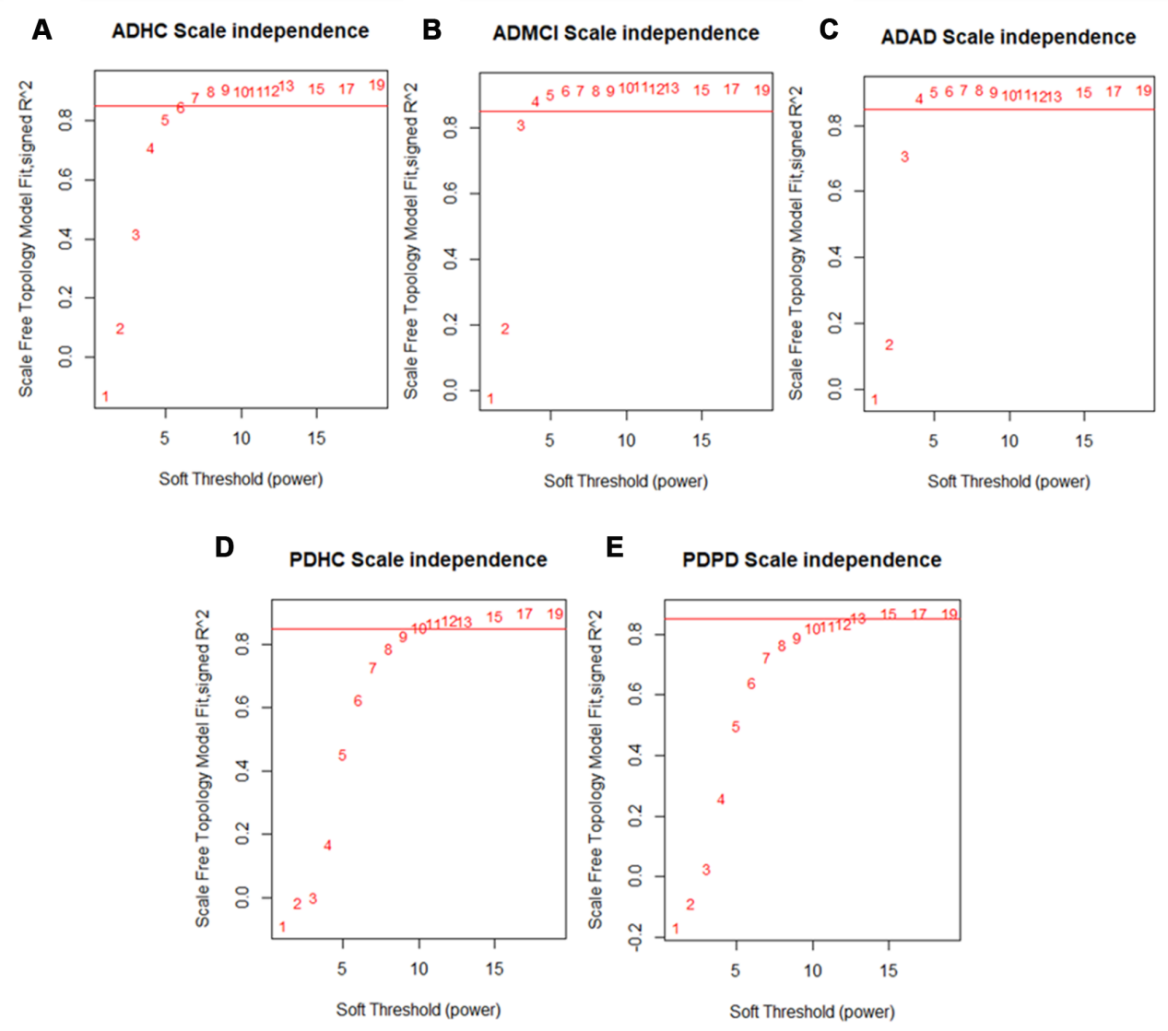
**Scale free network topology (signed R^2^) for different soft-thresholding powers of data.** A soft thresholding power that achieved a scale-free topology of R^2^ of 0.85 was chosen to define approximate scale-free topology. We found that the (**A**) ADHC data achieved approximate scale-free topology at a soft thresholding power of 6 and the (**B**) ADMCI and (**C**) ADAD data at a soft thresholding power of 4. The (**D**) PDHC data reached approximate scale-free topology at a soft thresholding power of 10 and (**E**) PDPD data at a soft thresholding power of 13.

After quality control, we obtained 19176 genes in the PD dataset which included 204 PD and 230 healthy control (HC) samples, meanwhile 13661 genes were obtained in the AD dataset which included 245 AD, 142 mild cognitive impairment (MCI) and 182 HC samples. We applied WGCNA [15] to build our networks and selected the soft threshold power to define the adjacency matrix of each dataset based on approximate scale-free topology R^2^ of 0.85 (Figure 2). In this method, highly correlated nodes are placed into a single module or cluster which are thought to be regulated by similar transcription factors (TFs) and represent certain biological processes. These networks were constructed for the AD disease (ADAD), healthy control (ADHC) and MCI (ADMCI) data from the AD dataset, and the PD disease (PDPD) and healthy control (PDHC) data from the PD dataset separately. We discovered 27, 54, 29, 32 and 58 modules in PDPD, PDHC, ADAD, ADMCI, ADHC networks respectively.

### PD blood and brain DEG overlap

We identified 360 DEGs in the PD blood dataset (nominal Pvalue < 0.01, see Supplementary Table 2) and compared these DEGs to the DEGs identified in our recent meta-analysis study about PD in substantia nigra region [11]. An overlap of 21 genes were found including *LRRN3*, *BASP1* and *TPM3*. However, a Fisher Exact test was not significant for the overlap showing that this was likely by chance (OR = 1.08, 95% CI 0.65~1.72, Pvalue = 0.72, Fisher Exact test).

### Identification of non-preserved modules

In our network analysis, if the relationships and correlation structure between nodes composing each module were not replicated, then they were considered non-preserved. In the case of healthy and disease networks, non-preserved modules suggested the expression pattern and regulation of the genes in these modules vary between disease and healthy conditions. On the other hand, modules preserved between disease and healthy networks represented processes that are not affected by disease status. Here we focused on non-preserved modules which may help to reveal the disease mechanism. The R package NetRep (v1.2.1) was used to identify these non-preserved modules [18].

Table 1 shows the non-preserved modules between PDHC and PDPD networks and the biological processes associated with these modules. Three of the 54 modules in the PDPD network were not preserved in PDHC network, and one of those 27 PDHC modules was not preserved in the PDPD network. The Gene Ontology (GO) and Kyoto Encyclopedia of Genes and Genomes (KEGG) terms that were significantly enriched within non-preserved modules (Pvalue < 0.01) were found using the Enrichr web tool [19, 20]. The PDPD salmon module was found to be associated with insulin signaling (KEGG pathway, Pvalue = 0.0030, 7/108 overlap). The PDPD darkseagreen4 module was found to be associated with antigen processing and presentation (KEGG pathway, Pvalue = 5.38E-16, overlap = 14/77) and natural killer cell mediated cytotoxicity (KEGG pathway, Pvalue = 2.94E-15, overlap = 10/41).

**Table 1:** List of non-preserved modules found between PD and healthy controls (HC).

**Module colour**	**Pvalue of NetRep**	**Processes associated with module found using Enrichr**	**No. genes in module**
*PD modules not preserved in HC*			
Darkseagreen4	9.99E-5	Antigen processing and presentation, Natural killer cell mediated cytotoxicity, cellular defense response, regulation of immune response	150
Navajowhite2	9.99E-5	cellular response to misfolded protein	150
Salmon	9.99E-5	Insulin resistance, regulation of protein homooligomerization	351
*HC modules not preserved in PD*			
Purple	9.99E-5	Antigen processing and presentation, VEGF signaling pathway, regulation of intracellular transport	606

Table 2 shows the non-preserved modules between the ADHC, ADMCI and ADAD networks. Of the 29 ADAD modules, one was not preserved in both ADHC and ADMCI networks. In addition, one of the 32 ADMCI modules was not preserved in ADAD and ADHC networks. Moreover, three of the 58 ADHC modules were not preserved in both ADAD and ADMCI networks and one non-preserved in ADMCI networks. The ADAD blue module was not preserved in ADHC and ADMCI networks and was associated with regulation of lipolysis in adipocytes (KEGG pathway, Pvalue = 6.24E-4, overlap = 10/55) and neuroactive ligand-receptor interaction (KEGG pathway, Pvalue = 0.005070, overlap = 30/338). The ADHC darkolivegreen module was associated with sensory perception (GO biological process, Pvalue = 1.83E-4, overlap = 8/55).

**Table 2:** List of non-preserved modules found between AD, MCI and healthy controls (HC).

**Module colour**	**Pvalue of NetRep**	**Processes associated with module found using Enrichr**	**No. genes in module**
*AD modules not preserved in HC*			
Blue	9.99E-5	Regulation of lipolysis in adipocytes, Neuroactive ligand-receptor interaction, detection of chemical stimulus involved in sensory perception of smell, extracellular matrix organization	1076
*AD modules not preserved in MCI*			
Blue	9.99E-5	Regulation of lipolysis in adipocytes, Neuroactive ligand-receptor interaction, detection of chemical stimulus involved in sensory perception of smell, extracellular matrix organization	1076
*MCI modules not preserved in AD*			
Sienna3	8.59E-3	Regulation of lipolysis in adipocytes, axonal fasciculation, hippo signaling	770
*MCI modules not preserved in HC*			
Sienna3	9.99E-5	Regulation of lipolysis in adipocytes, axonal fasciculation, hippo signaling	770
*HC modules not preserved in AD*			
Darkolivegreen	9.99E-5	sensory perception, regulation of potassium ion transmembrane transport	584
Darkorange2	0.011	Peroxisome, amide transport	248
Skyblue	0.015	establishment of epithelial cell polarity	187
*HC modules not preserved in MCI*			
Darkolivegreen	9.99E-5	sensory perception, regulation of potassium ion transmembrane transport	584
Red	9.99E-5	Regulation of lipolysis in adipocytes, bicellular tight junction assembly	704
Darkorange2	2.99E-4	Peroxisome, amide transport	248
Skyblue	0.022	establishment of epithelial cell polarity	187

### Identifying hub genes

Hubs are genes that are highly interconnected or important within a module and likely have functional significance [21]. Hubs have a role in maintaining the structure of the gene network of the module and the biological processes associated with the module. In our study, hub genes were identified using five approaches: Betweenness centrality (BC), PageRank, module membership (MM), closeness centrality and Kleinberg’s centrality. Any gene with a Pvalue < 0.01 in any hub detection method was considered as a significant hub gene. Using multiple methods for identifying hubs allowed for hub identification that may otherwise have been missed by use of just one method. To demonstrate hub score distribution, Supplementary Figure 2A shows an example of betweenness hub score distribution across all genes in the PDPD darkseagreen4 module which was non-preserved in PDHC network and the (Supplementary Figure 2B) distribution of the significant *GINS2* (Pvalue = 0.005) BC scores across the 1000 iterations of the hub permutation test.

**Figure 3:**
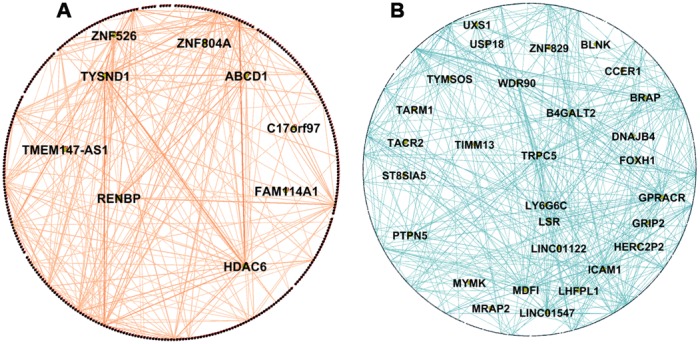
**Network visualization of PD and AD modules.** (**A**) Visualization of WGCNA network connections of the PDPD salmon network module found to be associated with insulin resistance and not preserved in the PDHC network. It shows network connections whose adjacency is above 0.2, including all 351 nodes and 595 of 61776 edges. (**B**) Visualization of WGCNA network connections of the ADAD blue module found to be associated with regulation of lipolysis in adipocytes and neuroactive ligand-receptor interaction and not preserved in ADHC and ADMCI networks. It shows network connections whose adjacency is above 0.55, including all 1076 nodes and 1458 of 1157776 edges. Hub genes are in the center of the network and are labelled with names. Networks visualized in Gephi [23].

We identified 34 hubs in modules not preserved between the PDPD and PDHC networks (Supplementary Table 3) and 92 hubs in the non-preserved modules between ADAD, ADMCI and ADHC networks (Supplementary Table 4). It was expected that larger modules may have more hubs than smaller ones, for example the PDHC purple module contained 606 genes, of which 17 were found to be hubs (e.g. *FAM110C*, *PAK4*, *NEB*), and the smaller salmon PDPD module contained 351 genes, of which only 10 were hubs (e.g. *HDAC6*, *TYSND1*). The PD salmon module was associated with insulin resistance and was not preserved in PDHC network shown in Figure 3A, where hub genes are highlighted. Interestingly, it includes *HDAC6* which has been shown to influence tau phosphorylation and autophagic flux in AD [22]. The blue AD module which was associated with regulation of lipolysis in adipocytes and neuroactive ligand-receptor interaction and was not preserved in ADMCI and ADHC networks (Figure 3B) which included *TRPC5* and *BRAP* as hub genes. Networks were visualized in Gephi [23].

### Identifying transcription factors (TFs)

Genes that are clustered together by WGNCA likely are regulated in a similar way, thus we intended to identify which TFs potentially regulate the gene expression of each module. The TFs that potentially regulate each non-preserved module (Pvalue <0.01) were identified by using Encyclopedia of DNA Elements (ENCODE) and chromatin immunoprecipitation (ChIP) Enrichment Analysis (ChEA) consensus TFs from ChIP-X by using the Enrichr web tool [19, 20]. We found a total of four TFs that regulated at least one of the three PDPD modules, including *FOXM1* which regulated 6 genes in the salmon modules (Pvalue = 0.0066) and 9 in the darkseagreen4 module (Pvalue = 4.00E-08). Within one PDHC module, there were a total of six TFs, including *CREB1* which regulated 64 genes in the purple module (Pvalue = 0.001402). Supplementary Table 5 shows the significant TFs found in modules that were not preserved between PD and HC networks.

We found two TFs (*SUZ12*, *EZH2*) regulating non-preserved ADAD modules, and one TF (*SUZ12*) regulating 115 genes in the ADMCI sienna3 module (Pvalue = 8.24E-10). We also identified 18 TFs that regulated at least one of four non-preserved ADHC modules. This included *REST* which regulated 20 genes in the darkolivegreen (Pvalue = 0.0092) and *SUZ12* which regulated 68 genes in the darkolivegreen (Pvalue = 0.0039) and 107 genes in the red module (Pvalue = 1.21E-09). In addition, *CREB1* regulated 29 genes in the ADHC darkorange2 module (Pvalue = 0.007005). Supplementary Table 6 shows the same for modules that were not preserved between ADAD, ADMCI and ADHC.

### Single nucleotide polymorphism (SNP) analysis of significant WGCNA modules

As non-preserved modules contain genes which play a role in processes that were associated with AD or PD, they may have been more likely to contain disease associated variants than preserved modules. We searched each non-preserved PD module for known Genome Wide Association Studies (GWAS) genes associated with PD [24]. There are 69 known GWAS genes, of which four (*TMEM163, TLR9, ITIH4, TUBG2*) were in the salmon module and two (*TMEM175, STAB1*) were in the navajowhite2 module. We observed a significant enrichment of GWAS genes within modules that were not preserved compared to preserved networks (OR = 2.96, 95% CI 1.04~6.88, Pvalue = 0.02, Fisher Exact test). Furthermore, the non-preserved PDHC purple network contained five GWAS gene (*KAT8, BIN3, TLR9, ITIH4, TUBG2*), however the non-preserved HC modules were not more likely to contain GWAS genes (OR = 2.61, 95% CI 0.08 ~ 6.47, Pvalue = 0.052, Fisher Exact test). We did the same analysis for the non-preserved AD modules, however, no AD associated GWAS genes were found within any non-preserved modules.

In addition to searching for known GWAS genes in non-preserved modules, we used the SCAN (SNP and Copy number ANnotation) database (http://www.scandb.org/) [25] to identify SNPs corresponding to the genes in each non-preserved module. These SNPs were used to search the MirSNP [26] database to identify SNPs associated with known PD or AD microRNAs (miRNAs) dependent on the dataset of the module. We identified 29 SNPs associated with 9 PD related miRNAs across all non-preserved modules in the PD dataset (Supplementary Table 7), and 27 SNPs associated with 8 AD related miRNAs across all non-preserved modules in the AD dataset (Supplementary Table 8).

### Comparison of AD and PD results

There is increasing evidence that PD and AD share several common characteristics [9], thus we investigated the shared processes associated with non-preserved modules in both the AD and PD dataset to see which were important in both diseases. The biological processes found to be associated with significant modules in AD and PD were compared to see which were important in both diseases. Unfortunately, we did not find any significant modules that were common between these two. However, we identified some similarities between AD and PD. The PDHC purple module and the ADHC darkorange2 module had four significant TFs which regulate both modules (*SIX5, CREB1, NFYB, PBX3*). Of those 29 PD SNPs and 27 AD SNPs we have identified, 12 were common between the two. The genes associated with these SNPs were: *EPB41L5, CYP26B1, IQCB1, DCP1A, CLGN, TDRD6, PSORS1C1, PARP12, WISP1, PIK3C2A, CLMN, DHX33* which are highlighted in Supplementary Tables 7 and 8.

### Data accession

The hub scores for each gene in PD modules not preserved in HC networks can be accessed and downloaded from https://jack-kelly.shinyapps.io/pdpd_hubs/. The same information for HC modules not preserved in PD networks can be found at https://jack-kelly.shinyapps.io/pdhc_hubs/.

The hub scores for each gene in the AD modules not preserved in HC or MCI networks can be found at https://jack-kelly.shinyapps.io/adad_hubs/. The same for MCI modules not preserved in HC or AD networks can be found at https://jack-kelly.shinyapps.io/admci_hubs/ and for HC modules not preserved in MCI or AD networks at https://jack-kelly.shinyapps.io/adhc_hubs/.

## DISCUSSION

In this study, by using gene co-expression network analysis we identified many important biological processes and key genes in PD and AD blood samples, and the common results between them. To our knowledge this is the largest network analysis of AD and PD blood to date. We found insulin resistance to be associated with PD and *HDAC6* may play an important role in this process. We highlight the overlap in disease miRNA associated SNPs that are shared between PD and AD, suggesting similarities in genetic risk factors between the diseases. Our approach used blood data as the available blood datasets have a large enough sample size to construct robust and reliable networks and blood samples are easily accessible in neurodegenerative disease patients. We previously found that DEGs in AD blood were more likely to be DEGs in AD brain tissue [27]. However, in this study, we found that DEGs in blood were not more likely to be DEGs in brain tissue for PD, nevertheless it has been shown that changes in blood gene expression did reflect changes in PD [28].

The PD network module associated with insulin resistance is not preserved in HCs. Insulin resistance is increasingly being shown to be important in PD as a potential therapeutic target [29] and has a high prevalence in non-diabetic PD patients [30], additionally insulin receptor signaling pathways are disturbed in PD [11]. Within this module we identified *HDAC6* as a hub gene which promotes the formation of inclusions from α-synuclein toxic oligomers [31]. *HDAC6* can promote insulin resistance by deacetylating phosphatase and tensin homolog (PTEN) in ovarian OVCAR-3 cells [32], and PTEN has in turn been shown to be involved in the pathophysiology of PD [33]. *HDAC6* has a role in influencing tau phosphorylation and autophagic flux in neurodegenerative disease [22]. In addition, insulin signaling promotes the DNA-binding activity of *FOXM1*, identified as a significant TF in the insulin resistance module, which regulates pathways to promote adaptive pancreatic β cell proliferation [34], but its role in ND is not clear.

The PD module associated with cellular response to misfolded proteins was also not preserved in HC networks. PD is characterized by accumulation of misfolded α-synuclein and a failure of the proteasome to degrade these and other large protein aggregates [35]. The hub gene *SNRNP70* has been shown to be differentially expressed in PD blood previously [36]. Additionally, *SNRNP70* encodes the small nuclear ribonucleoprotein snRNP70 which co-localizes with tau in AD [37], and as tau aggregation is shown in ~50% of PD cases snRNP70 may colocalize in PD cases [38]. We also identified *MIR142*, which encodes miRNA-142, as a hub. miRNA-142 has been identified as an important miRNA in PD, regulating GNAQ, TMTC2, BEND2, and KYNU [39].

The AD module associated with regulation of lipolysis in adipocytes and neuroactive ligand-receptor interaction was not preserved in both MCI and HC networks. Aβ, a key molecule in AD brain pathology, can induce lipolysis within human adipose tissue [40]. In addition, lipolysis is promoted by insulin resistance and in turn lipolysis generates ceramides further impairing insulin signaling, which is becoming increasingly more important in AD [41]. We identified *TRPC5* as a hub in this module, which along with other transient receptor potential canonical (TRPC) proteins assembles to form non-selective Ca2+-permeable channels. Another hub, *BRAP*, has a polymorphism associated with obesity and other metabolic traits, which can play a role in effecting insulin signaling and aging [42]. Interestingly, a module in the HC network that was not preserved in AD and MCI networks was also associated with regulation of lipolysis in adipocytes. This suggests that these processes are occurring in both healthy and AD conditions, however the enrichment pathways are different between the two. As no hubs are shared between the regulation of lipolysis in adipocytes modules in healthy and AD networks they are likely regulated differently.

The module associated with sensory perception in the HC network was not preserved in AD and MCI networks. Sensory dysfunction may precede the cognitive symptoms of AD [43], particularly olfactory impairment [44]. *OR5AS1* was identified as a hub gene within the module which encodes a member of the olfactory receptor family and plays a role in triggering response to smells [45]. The TF *REST* was identified as a regulator of the module and has been shown to regulate olfactory systems [46]. We have identified *REST* to be an important upstream TF for DEGs identified in both AD and PD previously, and as an important potential therapeutic target [11]. Future work to validate our identified hubs and TFs in both AD and PD disease models would further elucidate their potential as targets for disease treatment.

Although we did not identify any common non-preserved modules in the AD and PD cohorts, there were other similarities shared in the results. Four TFs were shared between the PDHC purple and the ADHC darkorange2 module (*CREB1, NFYB, PBX3, SIX5*). These two modules were associated with different transport pathways in HCs which were not preserved in the disease networks, suggesting that the roles of these TFs are dysregulated in both AD and PD. In addition to this, we identified 12 SNPs that were shared between the 29 PD miRNAs associated SNPs and 27 AD miRNAs associated SNPs. This number of shared SNPs is highly significant, which suggests that there are potential risk factors that underlie both diseases.

Several studies have applied WGCNA in ND studies for gene expression and proteomics analysis. For example, Seyfried and colleagues studied proteomic data of cortical tissue of asymptomatic and symptomatic AD [47]. They found that there was a modest overlap between networks at RNA and protein level. If a larger dataset becomes available, expanding our methods to proteomic data could give further understanding into the mechanisms of AD and PD and enable the investigation into the link between genomics and proteomics. Chatterjee et al. [17] have performed network analysis of PD brain tissue, however they only performed WGCNA on DEGs found in the data, which built very limited networks that removed potentially important gene interactions and disease regulators and introduced a bias of modules and hubs towards these DEGs. In addition, they used tissue from multiple brain regions which would all be affected differently by the disease [48].

A limitation of this study is that, although it has been shown that AD blood DEGs are more likely to be DEGs in the brain [27], our results suggest this is not the case for PD. Because of this, our results may not reflect major changes that take place in the brain. However, our network analysis approach emphasizes the interactions of genes which univariate methods like differential expression does not. Similarly to AD, there is disruption that happens in the blood brain barrier (BBB) of PD patients [49]. Hence, it is likely that changes that take place in the brain could be reflected in the blood and vice versa. Additionally, a lot of the biological processes and genes we found in our PD network has been implicated in the PD brain previously [11]. Tau and Aβ are hallmarks of both AD and PD in the brain and have potential as blood biomarkers in both diseases [50, 51], suggesting that changes in the brain are reflected in blood. Leukocytes have been shown to impact progression of neurodegenerative diseases. An interaction between brain and systemic inflammation has been implicated in PD progression by an association between leukocyte apoptosis and central dopamine neuron loss [55]. Increased mitochondrial respiratory activity in leukocytes has been shown in PD patients, potentially impacting progression of neurodegeneration [56] and elevated leukocytes in cerebrospinal fluid are significantly associated with shorter survival of patients [57]. Peripheral leukocytes have been discussed as potential biomarkers for AD previously [52], and gene expression changes in leukocytes have been shown to be closely associated with AD progression [53]. In AD animal models circulating leukocytes have been shown to cross a dysfunctional blood brain barrier and impact brain integrity [54].

Recently limbic-predominant age-related TDP-43 encephalopathy (LATE) has been reported to be under-recognized and often misdiagnosed as AD as they share common pathogenetic mechanisms and present similarly in patients [58]. There is the potential that patients in our AD cohort may have been misdiagnosed and actually have LATE, however as LATE is seen with increasing frequency over the age of 85, and less than 6% of our AD samples were over the age of 85 this likely had little effect on our results.

The greatest risk factor for both AD and PD is age. Adjusting AD data by age before WGCNA ensured any changes we found were reflective of disease state. The PD data, however, did not include samples’ age information when we downloaded, thus the effect of age could not be removed technically. As a result of this, the PD results may have been biased towards changes as a result of aging if there was a significant difference in age between PD and HC cohorts. However, the samples were age matched in the original design which should reduce such biases [59].

From the PD dataset we removed patient samples with known PD mutations. Although the biological pathways underlying familial and sporadic forms of PD are likely to be shared, known PD mutations may impact pathways to disease or regulators of disease [60]. Removal of samples with known PD mutations prevented these mutations from having an impact on results, however had little impact on sample size due to the low number of samples with mutations. AD samples were not screened for known mutations, which could have had an impact on our results. For example, nearly 19% of the familial late onset AD population carry 2 APOE ε4 alleles which only occurs in about 1% of normal Caucasian controls [61]. This and other known mutations may impact the progression and regulators of AD, and knowing which samples had these mutations could have improved our findings.

In conclusion, our network analysis is the largest study using AD and PD blood data to date. We highlight the non-preserved module in PD associated with insulin resistance, and the hub *HDAC6* identified in this module. Our results reveal that a large proportion of disease miRNA associated SNPs are shared between PD and AD, suggesting similarities in genetic risk factors between the diseases. The hub genes that we have identified have the possibility to be further investigated as potential biomarkers for disease. These insights suggest several new areas for mechanistic studies in PD and AD research fields.

## MATERIALS AND METHODS

### Data preparation for PD and AD blood datasets

The publicly available peripheral venous whole blood dataset comprising 205 PD and 233 control samples was downloaded from the GEO (Gene Expression Omnibus) database (http://www.ncbi.nlm.nih.gov/geo/) with accession identifier GSE99039. This dataset is the largest of its type and has a sample size enough to run WGCNA and reliably find hub genes [62]. Samples with known PD mutation genes (*Parkin, DJ-1* and *PINK1, ATP13A2, LRRK2, SNCA*) were removed to reduce biases introduced by these genes (see discussion), and outlier samples were detected and removed based on box and density plots of probe intensities. This removed a total of one PD and three HC samples, leaving 204 PD and 230 HC samples. Data was then Robust Multiarray Average (RMA) normalized using the affy R package [63]. Samples missing gender information (35 samples) were assigned sex by using the massiR R package [64] which uses the information from microarray probes that represent genes in Y chromosome to perform k-medoids clustering to classify the samples into male and female groups. We selected a probe-variation threshold of 4 by inspecting a probe-variation plot (Supplementary Figure 1) to select the Y chromosome probes to be used in the sex classification process.

The ComBat function in the sva R package [65] was used to control the effect of gender and running batch of the samples. After this, control probes and those without Entrez gene annotation were removed. For any genes that mapped to multiple probes, the probe with the highest median absolute deviation (MAD) was kept. MAD was used as, similarly to inter-quartile range, the probe with the highest MAD has the greatest variability and so likely has more information [66]. Finally, the bottom 5% probes by average expression values across all samples were removed.

For AD, the two independent peripheral venous whole blood datasets GSE63060 and GSE63061, from the AddNeuroMed Cohort [67], were used to construct the blood gene expression networks. As these two datasets were from the same cohort study and sample collection and analysis was carried out using the same methodologies, except using different biological samples and microarray platforms, they can be merged to produce a larger dataset that can improve the power of our study. The two normalized datasets (generated by different Illumina platforms) were merged using the inSilicoMerging R package [68], which removes the batch effects between these two, as we have done previously [27].

Patients of Western European and Caucasian ethnicity were extracted from the merged dataset leaving a total of 245 AD, 142 MCI and 182 HC to reduce any potential genetic impact that ethnicity may have on AD. The effect of the age and gender were controlled for using the ComBat function in the sva R package [65]. As with the PD data, control probes and those without Entrez gene annotation were removed and for any genes that mapped to multiple probes, the probe with the highest MAD was kept. Finally, the bottom 5% probes by average expression values across samples were removed. Information on number of samples, gender and age of samples is shown in Supplementary Table 1.

### PD blood and brain DEG overlap

To see if there was a significant overlap between PD gene expression in blood and brain as has been shown previously in AD [27], our data was compared to DEGs previously identified in PD substantia nigra [11]. Using the normalized and filtered PD data, DEGs were identified by applying limma with gender and running batch adjusted. Slightly stringent nominal Pvalue <0.01 was used for significance as only one DEG could pass multiple testing (FDR corrected Pvalue <0.05).

### Gene co-expression network construction

The R package WGCNA [15] was applied to perform gene co-expression network analysis as follows: A matrix of pairwise correlations between all pairs of genes across each sample group (e.g. case and control groups separately), was created and each raised to a soft-thresholding power to achieve a scale-free topology R^2^ of 0.85. From this, a topological overlap matrix (TOM) was calculated, which takes correlation between genes expression as well as connections the genes share into consideration. This TOM was then converted to topological overlap dissimilarities to be used with hierarchical clustering. Then, a dynamic tree-cutting algorithm was used to determine initial module assignments of genes (cutreeHybrid, using default parameters except deepSplit of 3, minModuleSize of 10 and mergeCutHeight of 0.05) [69]. An additional *k*-means clustering step was applied to improve the results of the hierarchical clustering in WGCNA as proposed by Botía et al [70] which has been reported to be able to reduce the number of misplaced genes and improve the enrichment of GO pathway terms. All analysis was conducted in R3.5.2 [71].

### Calculation of module preservation

In order to identify modules that are not preserved between conditions within datasets, we applied NetRep (v1.2.1) [18] which uses a permutation test procedure on seven module preservation statistics. We permuted 10,000 times. The “alternative” parameter is set to “less” to test whether each module preservation statistic is smaller than expected by chance in order to identify these non-preserved modules which are extremely different in the two networks. If all seven module preservation statistics had a Pvalue < 0.05 then that module was significantly non-preserved between conditions.

### Pathway enrichment analysis

To identify the biological pathways that the modules represent we performed GO and KEGG pathway enrichment analysis (KEGG 2019) using the Enrichr web tool [19, 20]. Pathways and GO terms with a Pvalue < 0.05 were considered significant.

### Hub gene identification

Generally, detecting hub genes in co-expression networks has been done using MM, which is the correlation of a gene to its eigengene (the first principle component calculated using the expression data of genes in each module) [72]. BC of a gene is the number of shortest paths connecting all gene pairs that pass through that gene [73], and genes with high BC were considered as “high traffic”.

Here we have expanded hub detection to include multiple other hub detection methods frequently used in network analysis. In addition to MM and BC, we used closeness centrality [74], Kleinberg's hub centrality score [75] and the PageRank algorithm [76] which would reduce the chance of missing any important hub genes that regulate the network that may be missed by applying individual methods. Genes with high closeness centrality scores have the shortest path to all other genes in the module and are placed to influence the entire network quickly [74]. PageRank emphasizes nodes that are connected to other nodes with high Pagerank scores [76]. Kleinberg's hub centrality score [75] is similar to the PageRank algorithm, however, the small differences between the two widens the net for identifying important hubs.

A novel hub detection permutation test was developed to obtain Pvalues for each hub detection store and determine if they are statistically significant. Briefly, the gene ID labels on the adjacency matrix were randomly re-labelled and hub score recalculated 1000 times to obtain a statistical distribution. The Pvalue was calculated by dividing the number of recalculated permutation hub scores that are higher than the observed hub score in the original network by the number of permutations. Genes were considered significant hubs if any hub scores had a Pvalue < 0.01. This was performed for all modules not preserved between PD and HCs in the PD dataset, and the modules not preserved between any of the AD, MCI and HCs networks in the AD dataset. BC, closeness centrality, PageRank and Kleinberg's hub centrality scores were calculated using the igraph R package with default settings without normalization [77]. The R code used for the novel hub detection test is available at http://dx.doi.org/10.5281/zenodo.3686007.

### Identifying transcription factors

To identify TFs that potentially regulate each module, we used ENCODE and ChEA Consensus TFs from ChIP-X found using the Enrichr web tool [19, 20]. TFs with a Pvalue < 0.01 were considered significant. If a TF was found significant in both ENCODE and ChEA then the lower Pvalue was assigned to the TF.

### SNP and microRNA analysis of significant WGCNA modules

A two-tailed Fisher’s exact test was used to test our hypothesis that non-preserved modules were more likely to contain GWAS identified genes than preserved modules. The risk loci for PD and AD were from recent GWAS, between which only one GWAS gene was shared (*KAT8*) [24, 78].

We gained further insight into SNPs associated with non-preserved modules, using a similar methodology to Chatterjee et al. [17]. The SCAN database [25] was used to find all SNPs that have been shown to predict the expression of each gene within non-preserved modules. For each non-preserved module, only SNPs that predicted gene expression with Pvalues < 1.0e-4 and frequency > 0.10 within the CEU human samples of European descent were selected.

Previous studies have revealed that differential expression of miRNAs were associated with PD [79] and AD [80]. In addition, SNPs have been identified as disease prognostic markers by association to miRNAs [81]. SNPs we found to be associated with genes from the PD related modules were used to search the MirSNP [26] database in order to find which SNPs were associated with the 83 experimentally confirmed PD related miRNAs in the HMDD v3.0 database [82]. The same process was done for genes within the AD related modules and the 57 experimentally confirmed AD related miRNAs in the HMDD v3.0 database. The MirSNP database identified the SNPs that are present at the 3' untranslated region of miRNA target sites, and so narrowed down the selection of SNPs to those that likely effect known miRNAs associated with the disease.

### Comparison of PD and AD results

The processes associated with non-preserved modules in AD and PD were compared to see if any processes were similar between diseases. Hub genes and TFs identified in non-preserved modules were also compared between AD and PD to see if any were shared. In addition, we test our hypothesis that AD and PD share SNPs we identified in non-preserved modules associated with disease related miRNAs in AD and PD respectively.

## Supplementary Material

Supplementary Figures

Supplementary Tables 1, 3, 5-8

Supplementary Table 2

Supplementary Table 4

## References

[1] Cacace R, Sleegers K, Van Broeckhoven C. Molecular genetics of early-onset Alzheimer's disease revisited. Alzheimers Dement. 2016; 12:733–48. 10.1016/j.jalz.2016.01.012 27016693

[2] Alzheimer’s Association. 2018 Alzheimer’s Disease Facts and Figures. 2018 https://www.alz.org/

[3] Shi L, Baird AL, Westwood S, Hye A, Dobson R, Thambisetty M, Lovestone S. A Decade of Blood Biomarkers for Alzheimer’s Disease Research: An Evolving sField, Improving Study Designs, and the Challenge of Replication. J Alzheimers Dis. 2018; 62:1181–98. 10.3233/JAD-170531 29562526 PMC5870012

[4] Long J, Pan G, Ifeachor E, Belshaw R, Li X. Discovery of Novel Biomarkers for Alzheimer’s Disease from Blood. Dis Markers. 2016; 2016:4250480. 10.1155/2016/4250480 27418712 PMC4932164

[5] Li X, Long J, He T, Belshaw R, Scott J. Integrated genomic approaches identify major pathways and upstream regulators in late onset Alzheimer’s disease. Sci Rep. 2015; 5:12393. 10.1038/srep12393 26202100 PMC4511863

[6] Parkinson’s UK. The prevalence and incidence of Parkinson’s in the UK. London; 2017.

[7] Marras C, Beck JC, Bower JH, Roberts E, Ritz B, Ross GW, Abbott RD, Savica R, Van Den Eeden SK, Willis AW, Tanner CM; Parkinson’s Foundation P4 Group. Prevalence of Parkinson's disease across North America. NPJ Parkinsons Dis. 2018; 4:21. 10.1038/s41531-018-0058-0 30003140 PMC6039505

[8] Kalinderi K, Bostantjopoulou S, Fidani L. The genetic background of Parkinson’s disease: current progress and future prospects. Acta Neurol Scand. 2016; 134:314–26. 10.1111/ane.12563 26869347

[9] Xie A, Gao J, Xu L, Meng D. Shared mechanisms of neurodegeneration in Alzheimer's disease and Parkinson's disease. Biomed Res Int. 2014; 2014:648740. 10.1155/2014/648740 24900975 PMC4037122

[10] Anang JB, Nomura T, Romenets SR, Nakashima K, Gagnon JF, Postuma RB. Dementia Predictors in Parkinson Disease: A Validation Study. J Parkinsons Dis. 2017; 7:159–62. 10.3233/JPD-160925 27911340

[11] Kelly J, Moyeed R, Carroll C, Albani D, Li X. Gene expression meta-analysis of Parkinson’s disease and its relationship with Alzheimer’s disease. Mol Brain. 2019; 12:16. 10.1186/s13041-019-0436-5 30819229 PMC6396547

[12] Kaźmierczak A, Czapski GA, Adamczyk A, Gajkowska B, Strosznajder JB. A novel mechanism of non-Aβ component of Alzheimer’s disease amyloid (NAC) neurotoxicity. Interplay between p53 protein and cyclin-dependent kinase 5 (Cdk5). Neurochem Int. 2011; 58:206–14. 10.1016/j.neuint.2010.11.018 21130128

[13] Kwon OD. Is There Any Relationship between Apolipoprotein E Polymorphism and Idiopathic Parkinson’s Disease? J Alzheimer’s Dis Park. 2017; 7:292 10.4172/2161-0460.1000296

[14] Miller JA, Oldham MC, Geschwind DH. A systems level analysis of transcriptional changes in Alzheimer’s disease and normal aging. J Neurosci. 2008; 28:1410–20. 10.1523/JNEUROSCI.4098-07.2008 18256261 PMC2902235

[15] Langfelder P, Horvath S. WGCNA: an R package for weighted correlation network analysis. BMC Bioinformatics. 2008; 9:559. 10.1186/1471-2105-9-559 19114008 PMC2631488

[16] Lunnon K, Ibrahim Z, Proitsi P, Lourdusamy A, Newhouse S, Sattlecker M, Furney S, Saleem M, Soininen H, Kłoszewska I, Mecocci P, Tsolaki M, Vellas B, et al, and AddNeuroMed Consortium. Mitochondrial dysfunction and immune activation are detectable in early Alzheimer’s disease blood. J Alzheimers Dis. 2012; 30:685–710. 10.3233/JAD-2012-111592 22466004

[17] Chatterjee P, Roy D, Bhattacharyya M, Bandyopadhyay S. Biological networks in Parkinson’s disease: an insight into the epigenetic mechanisms associated with this disease. BMC Genomics. 2017; 18:721. 10.1186/s12864-017-4098-3 28899360 PMC5596942

[18] Ritchie SC, Watts S, Fearnley LG, Holt KE, Abraham G, Inouye M. A Scalable Permutation Approach Reveals Replication and Preservation Patterns of Network Modules in Large Datasets. Cell Syst. 2016; 3:71–82. 10.1016/j.cels.2016.06.012 27467248

[19] Chen EY, Tan CM, Kou Y, Duan Q, Wang Z, Meirelles GV, Clark NR, Ma’ayan A. Enrichr: interactive and collaborative HTML5 gene list enrichment analysis tool. BMC Bioinformatics. 2013; 14:128. 10.1186/1471-2105-14-128 23586463 PMC3637064

[20] Kuleshov MV, Jones MR, Rouillard AD, Fernandez NF, Duan Q, Wang Z, Koplev S, Jenkins SL, Jagodnik KM, Lachmann A, McDermott MG, Monteiro CD, Gundersen GW, Ma’ayan A. Enrichr: a comprehensive gene set enrichment analysis web server 2016 update. Nucleic Acids Res. 2016; 44:W90–7. 10.1093/nar/gkw377 27141961 PMC4987924

[21] Albert R. Scale-free networks in cell biology. J Cell Sci. 2005; 118:4947–57. 10.1242/jcs.02714 16254242

[22] Richter-Landsberg C, Leyk J. Inclusion body formation, macroautophagy, and the role of HDAC6 in neurodegeneration. Acta Neuropathol. 2013; 126:793–807. 10.1007/s00401-013-1158-x 23912309

[23] Bastian M, Heymann S, Jacomy M. Gephi: An open source software for exploring and manipulating networks. International AAAI Conference on Weblogs and Social Media. 2009.

[24] Chang D, Nalls MA, Hallgrímsdóttir IB, Hunkapiller J, van der Brug M, Cai F, Kerchner GA, Ayalon G, Bingol B, Sheng M, Hinds D, Behrens TW, Singleton AB, et al, and International Parkinson’s Disease Genomics Consortium, and 23andMe Research Team. A meta-analysis of genome-wide association studies identifies 17 new Parkinson’s disease risk loci. Nat Genet. 2017; 49:1511–16. 10.1038/ng.3955 28892059 PMC5812477

[25] Gamazon ER, Zhang W, Konkashbaev A, Duan S, Kistner EO, Nicolae DL, Dolan ME, Cox NJ. SCAN: SNP and copy number annotation. Bioinformatics. 2010; 26:259–62. 10.1093/bioinformatics/btp644 19933162 PMC2852202

[26] Liu C, Zhang F, Li T, Lu M, Wang L, Yue W, Zhang D. MirSNP, a database of polymorphisms altering miRNA target sites, identifies miRNA-related SNPs in GWAS SNPs and eQTLs. BMC Genomics. 2012; 13:661. 10.1186/1471-2164-13-661 23173617 PMC3582533

[27] Li X, Wang H, Long J, Pan G, He T, Anichtchik O, Belshaw R, Albani D, Edison P, Green EK, Scott J. Systematic Analysis and Biomarker Study for Alzheimer's Disease. Sci Rep. 2018; 8:17394. 10.1038/s41598-018-35789-3 30478411 PMC6255913

[28] Pinho R, Guedes LC, Soreq L, Lobo PP, Mestre T, Coelho M, Rosa MM, Gonçalves N, Wales P, Mendes T, Gerhardt E, Fahlbusch C, Bonifati V, et al. Gene expression differences in peripheral blood of Parkinson’s disease patients with distinct progression profiles. PLoS One. 2016; 11:e0157852. 10.1371/journal.pone.0157852 27322389 PMC4913914

[29] Vidal-Martinez G, Yang B, Vargas-Medrano J, Perez RG. Could α-synuclein modulation of insulin and dopamine identify a novel link between parkinson’s disease and diabetes as well as potential therapies? Front Mol Neurosci. 2018; 11:465. 10.3389/fnmol.2018.00465 30622456 PMC6308185

[30] Hogg E, Athreya K, Basile C, Tan EE, Kaminski J, Tagliati M. High prevalence of undiagnosed insulin resistance in non-diabetic subjects with Parkinson’s disease. J Parkinsons Dis. 2018; 8:259–65. 10.3233/JPD-181305 29614702

[31] Simões-Pires C, Zwick V, Nurisso A, Schenker E, Carrupt PA, Cuendet M. HDAC6 as a target for neurodegenerative diseases: what makes it different from the other HDACs? Mol Neurodegener. 2013; 8:7. 10.1186/1750-1326-8-7 23356410 PMC3615964

[32] Qu X, Huang C, Qu H, Jia B, Cui Q, Sun C, Chu Y. Histone deacetylase 6 promotes insulin resistance via deacetylating phosphatase and tensin homolog (PTEN) in ovarian OVCAR-3 cells. Int J Clin Exp Pathol. 2016; 9:7105–13.

[33] Sekar S, Taghibiglou C. Elevated nuclear phosphatase and tensin homolog (PTEN) and altered insulin signaling in substantia nigral region of patients with Parkinson’s disease. Neurosci Lett. 2018; 666:139–43. 10.1016/j.neulet.2017.12.049 29288045

[34] Shirakawa J, Fernandez M, Takatani T, El Ouaamari A, Jungtrakoon P, Okawa ER, Zhang W, Yi P, Doria A, Kulkarni RN. Insulin signaling regulates the FoxM1/PLK1/CENP-A pathway to promote adaptive pancreatic β-cell proliferation. Cell Metab. 2017; 25:868–882.e5. 10.1016/j.cmet.2017.02.004 28286049 PMC5382039

[35] Lehtonen Š, Sonninen TM, Wojciechowski S, Goldsteins G, Koistinaho J. Dysfunction of cellular proteostasis in Parkinson’s disease. Front Neurosci. 2019; 13:457. 10.3389/fnins.2019.00457 31133790 PMC6524622

[36] Santiago JA, Potashkin JA. Blood transcriptomic meta-analysis identifies dysregulation of hemoglobin and iron metabolism in Parkinson’ disease. Front Aging Neurosci. 2017; 9:73. 10.3389/fnagi.2017.00073 28424608 PMC5372821

[37] Diner I, Hales CM, Bishof I, Rabenold L, Duong DM, Yi H, Laur O, Gearing M, Troncoso J, Thambisetty M, Lah JJ, Levey AI, Seyfried NT. Aggregation properties of the small nuclear ribonucleoprotein U1-70K in Alzheimer disease. J Biol Chem. 2014; 289:35296–313. 10.1074/jbc.M114.562959 25355317 PMC4271217

[38] Zhang X, Gao F, Wang D, Li C, Fu Y, He W, Zhang J. Tau pathology in Parkinson’s disease. Front Neurol. 2018; 9:809. 10.3389/fneur.2018.00809 30333786 PMC6176019

[39] Liu X, Chen J, Guan T, Yao H, Zhang W, Guan Z, Wang Y. miRNAs and target genes in the blood as biomarkers for the early diagnosis of Parkinson’s disease. BMC Syst Biol. 2019; 13:10. 10.1186/s12918-019-0680-4 30665415 PMC6341689

[40] Wan Z, Mah D, Simtchouk S, Kluftinger A, Little JP. Role of amyloid β in the induction of lipolysis and secretion of adipokines from human adipose tissue. Adipocyte. 2014; 4:212–16. 10.4161/21623945.2014.985020 26257989 PMC4497301

[41] Ferreira LS, Fernandes CS, Vieira MN, De Felice FG. Insulin Resistance in Alzheimer’s Disease. Front Neurosci. 2018; 12:830. 10.3389/fnins.2018.00830 30542257 PMC6277874

[42] Imaizumi T, Ando M, Nakatochi M, Yasuda Y, Honda H, Kuwatsuka Y, Kato S, Kondo T, Iwata M, Nakashima T, Yasui H, Takamatsu H, Okajima H, et al. Effect of dietary energy and polymorphisms in BRAP and GHRL on obesity and metabolic traits. Obes Res Clin Pract. 2018 (Suppl 2); 12:39–48. 10.1016/j.orcp.2016.05.004 27245511

[43] Albers MW, Gilmore GC, Kaye J, Murphy C, Wingfield A, Bennett DA, Boxer AL, Buchman AS, Cruickshanks KJ, Devanand DP, Duffy CJ, Gall CM, Gates GA, et al. At the interface of sensory and motor dysfunctions and Alzheimer’s disease. Alzheimers Dement. 2015; 11:70–98. 10.1016/j.jalz.2014.04.514 25022540 PMC4287457

[44] Murphy C. Olfactory and other sensory impairments in Alzheimer disease. Nat Rev Neurol. 2019; 15:11–24. 10.1038/s41582-018-0097-5 30532084

[45] Malnic B, Godfrey PA, Buck LB. The human olfactory receptor gene family. Proc Natl Acad Sci USA. 2004; 101:2584–89. 10.1073/pnas.0307882100 14983052 PMC356993

[46] Casadei E, Tacchi L, Lickwar CR, Espenschied ST, Davison JM, Muñoz P, Rawls JF, Salinas I. Commensal Bacteria Regulate Gene Expression and Differentiation in Vertebrate Olfactory Systems Through Transcription Factor REST. Chem Senses. 2019; 44:615–30. 10.1093/chemse/bjz050 31403159 PMC6796929

[47] Seyfried NT, Dammer EB, Swarup V, Nandakumar D, Duong DM, Yin L, Deng Q, Nguyen T, Hales CM, Wingo T, Glass J, Gearing M, Thambisetty M, et al. A Multi-network Approach Identifies Protein-Specific Co-expression in Asymptomatic and Symptomatic Alzheimer’s Disease. Cell Syst. 2017; 4:60–72.e4. 10.1016/j.cels.2016.11.006 27989508 PMC5269514

[48] Poewe W, Seppi K, Tanner CM, Halliday GM, Brundin P, Volkmann J, Schrag AE, Lang AE. Parkinson disease. Nat Rev Dis Primers. 2017; 3:17013. 10.1038/nrdp.2017.13 28332488

[49] Gray MT, Woulfe JM. Striatal blood-brain barrier permeability in Parkinson’s disease. J Cereb Blood Flow Metab. 2015; 35:747–50. 10.1038/jcbfm.2015.32 25757748 PMC4420870

[50] Lue LF, Guerra A, Walker DG. Amyloid Beta and Tau as Alzheimer’s Disease Blood Biomarkers: Promise From New Technologies. Neurol Ther. 2017 (Suppl 1); 6:25–36. 10.1007/s40120-017-0074-8 28733956 PMC5520820

[51] Chojdak-Łukasiewicz J, Małodobra-Mazur M, Zimny A, Noga L, Paradowski B. Plasma tau protein and Aβ42 level as markers of cognitive impairment in patients with Parkinson’s disease. Adv Clin Exp Med. 2020; 29:115–21. 10.17219/acem/112058 31990459

[52] Rezai-Zadeh K, Gate D, Szekely CA, Town T. Can peripheral leukocytes be used as Alzheimer’s disease biomarkers? Expert Rev Neurother. 2009; 9:1623–33. 10.1586/ern.09.118 19903022 PMC2828773

[53] Li H, Hong G, Lin M, Shi Y, Wang L, Jiang F, Zhang F, Wang Y, Guo Z. Identification of molecular alterations in leukocytes from gene expression profiles of peripheral whole blood of Alzheimer’s disease. Sci Rep. 2017; 7:14027. 10.1038/s41598-017-13700-w 29070791 PMC5656592

[54] Cai Z, Qiao PF, Wan CQ, Cai M, Zhou NK, Li Q. Role of Blood-Brain Barrier in Alzheimer’s Disease. J Alzheimers Dis. 2018; 63:1223–34. 10.3233/JAD-180098 29782323

[55] Lin WC, Tsai NW, Huang YC, Cheng KY, Chen HL, Li SH, Kung CT, Su YJ, Lin WM, Chen MH, Chiu TM, Yang IH, Lu CH. Peripheral leukocyte apoptosis in patients with Parkinsonism: correlation with clinical characteristics and neuroimaging findings. Biomed Res Int. 2014; 2014:635923. 10.1155/2014/635923 24795890 PMC3984850

[56] Annesley SJ, Lay ST, De Piazza SW, Sanislav O, Hammersley E, Allan CY, Francione LM, Bui MQ, Chen ZP, Ngoei KR, Tassone F, Kemp BE, Storey E, et al. Immortalized Parkinson’s disease lymphocytes have enhanced mitochondrial respiratory activity. Dis Model Mech. 2016; 9:1295–305. 10.1242/dmm.025684 27638668 PMC5117226

[57] Bäckström D, Granåsen G, Domellöf ME, Linder J, Jakobson Mo S, Riklund K, Zetterberg H, Blennow K, Forsgren L. Early predictors of mortality in parkinsonism and Parkinson disease: A population-based study. Neurology. 2018; 91:e2045–56. 10.1212/WNL.0000000000006576 30381367 PMC6282235

[58] Nelson PT, Dickson DW, Trojanowski JQ, Jack CR, Boyle PA, Arfanakis K, Rademakers R, Alafuzoff I, Attems J, Brayne C, Coyle-Gilchrist IT, Chui HC, Fardo DW, et al. Limbic-predominant age-related TDP-43 encephalopathy (LATE): consensus working group report. Brain. 2019; 142:1503–27. 10.1093/brain/awz099 31039256 PMC6536849

[59] Shamir R, Klein C, Amar D, Vollstedt EJ, Bonin M, Usenovic M, Wong YC, Maver A, Poths S, Safer H, Corvol JC, Lesage S, Lavi O, et al. Analysis of blood-based gene expression in idiopathic Parkinson disease. Neurology. 2017; 89:1676–83. 10.1212/WNL.0000000000004516 28916538 PMC5644465

[60] Chai C, Lim KL. Genetic insights into sporadic Parkinson’s disease pathogenesis. Curr Genomics. 2013; 14:486–501. 10.2174/1389202914666131210195808 24532982 PMC3924245

[61] Bekris LM, Yu CE, Bird TD, Tsuang DW. Genetics of Alzheimer disease. J Geriatr Psychiatry Neurol. 2010; 23:213–27. 10.1177/0891988710383571 21045163 PMC3044597

[62] Allen JD, Xie Y, Chen M, Girard L, Xiao G. Comparing statistical methods for constructing large scale gene networks. PLoS One. 2012; 7:e29348. 10.1371/journal.pone.0029348 22272232 PMC3260142

[63] Gautier L, Cope L, Bolstad BM, Irizarry RA. affy—analysis of Affymetrix GeneChip data at the probe level. Bioinformatics. 2004; 20:307–15. 10.1093/bioinformatics/btg405 14960456

[64] Buckberry S, Bent SJ, Bianco-Miotto T, Roberts CT, Massi R. massiR: a method for predicting the sex of samples in gene expression microarray datasets. Bioinformatics. 2014; 30:2084–85. 10.1093/bioinformatics/btu161 24659105 PMC4080740

[65] Leek JT, Johnson WE, Parker HS, Jaffe AE, Storey JD, Zhang Y. sva: Surrogate Variable Analysis. 2019.

[66] Wang X, Lin Y, Song C, Sibille E, Tseng GC. Detecting disease-associated genes with confounding variable adjustment and the impact on genomic meta-analysis: with application to major depressive disorder. BMC Bioinformatics. 2012; 13:52. 10.1186/1471-2105-13-52 22458711 PMC3342232

[67] Sood S, Gallagher IJ, Lunnon K, Rullman E, Keohane A, Crossland H, Phillips BE, Cederholm T, Jensen T, van Loon LJ, Lannfelt L, Kraus WE, Atherton PJ, et al. A novel multi-tissue RNA diagnostic of healthy ageing relates to cognitive health status. Genome Biol. 2015; 16:185. 10.1186/s13059-015-0750-x 26343147 PMC4561473

[68] Taminau J, Meganck S, Lazar C, Steenhoff D, Coletta A, Molter C, Duque R, de Schaetzen V, Weiss Solís DY, Bersini H, Nowé A. Unlocking the potential of publicly available microarray data using inSilicoDb and inSilicoMerging R/Bioconductor packages. BMC Bioinformatics. 2012; 13:335. 10.1186/1471-2105-13-335 23259851 PMC3568420

[69] Langfelder P, Zhang B, Horvath S. Defining clusters from a hierarchical cluster tree: the Dynamic Tree Cut package for R. Bioinformatics. 2008; 24:719–20. 10.1093/bioinformatics/btm563 18024473

[70] Botía JA, Vandrovcova J, Forabosco P, Guelfi S, D’Sa K, Hardy J, Lewis CM, Ryten M, Weale ME, and United Kingdom Brain Expression Consortium. An additional k-means clustering step improves the biological features of WGCNA gene co-expression networks. BMC Syst Biol. 2017; 11:47. 10.1186/s12918-017-0420-6 28403906 PMC5389000

[71] R Core Team. R: A language and environment for statistical computing. Vienna, Austria: R Foundation for Statistical Computing; 2017.

[72] Yuan L, Chen L, Qian K, Qian G, Wu CL, Wang X, Xiao Y. Co-expression network analysis identified six hub genes in association with progression and prognosis in human clear cell renal cell carcinoma (ccRCC). Genom Data. 2017; 14:132–140. 10.1016/j.gdata.2017.10.006 29159069 PMC5683669

[73] Brandes U. A faster algorithm for betweenness centrality. J Math Sociol. 2001; 25:163–77. 10.1080/0022250X.2001.9990249

[74] Newman MJ. A measure of betweenness centrality based on random walks. Soc Networks. 2005; 27:39–54. 10.1016/j.socnet.2004.11.009

[75] Kleinberg JM. Authoritative sources in a hyperlinked environment. J Assoc Comput Mach. 1999; 46:604–32. 10.1145/324133.324140

[76] Brin S, Page L. The anatomy of a large-scale hypertextual Web search engine. Comput Netw ISDN Syst. 1998; 30:107–17. 10.1016/S0169-7552(98)00110-X

[77] Csardi G, Nepusz T. The igraph software package for complex network research. Inter Journal. 2006; Complex Sy: 1695.

[78] Jansen IE, Savage JE, Watanabe K, Bryois J, Williams DM, Steinberg S, Sealock J, Karlsson IK, Hägg S, Athanasiu L, Voyle N, Proitsi P, Witoelar A, et al. Genome-wide meta-analysis identifies new loci and functional pathways influencing Alzheimer’s disease risk. Nat Genet. 2019; 51:404–13. 10.1038/s41588-018-0311-9 30617256 PMC6836675

[79] Martins M, Rosa A, Guedes LC, Fonseca BV, Gotovac K, Violante S, Mestre T, Coelho M, Rosa MM, Martin ER, Vance JM, Outeiro TF, Wang L, et al. Convergence of miRNA expression profiling, α-synuclein interacton and GWAS in Parkinson’s disease. PLoS One. 2011; 6:e25443. 10.1371/journal.pone.0025443 22003392 PMC3189215

[80] Zovoilis A, Agbemenyah HY, Agis-Balboa RC, Stilling RM, Edbauer D, Rao P, Farinelli L, Delalle I, Schmitt A, Falkai P, Bahari-Javan S, Burkhardt S, Sananbenesi F, Fischer A. microRNA-34c is a novel target to treat dementias. EMBO J. 2011; 30:4299–308. 10.1038/emboj.2011.327 21946562 PMC3199394

[81] Guo Z, Wang H, Li Y, Li B, Li C, Ding C. A microRNA-related single nucleotide polymorphism of the *XPO5* gene is associated with survival of small cell lung cancer patients. Biomed Rep. 2013; 1:545–48. 10.3892/br.2013.92 24648983 PMC3917003

[82] Huang Z, Shi J, Gao Y, Cui C, Zhang S, Li J, Zhou Y, Cui Q. HMDD v3.0: a database for experimentally supported human microRNA-disease associations. Nucleic Acids Res. 2019; 47:D1013–D1017. 10.1093/nar/gky1010 30364956 PMC6323994

