# Supplementary material for: Genetic networks in Parkinson’s and Alzheimer’s disease
Source: Aging (Albany NY). 2020 Mar 23;12(6):5221–43. doi: 10.18632/aging.102943 (PMC7138567; doi:10.18632/aging.102943)
Supplement: Supplementary Figures [file aging-12-102943-s004..pdf]

SUPPLEMENTARY FIGURES

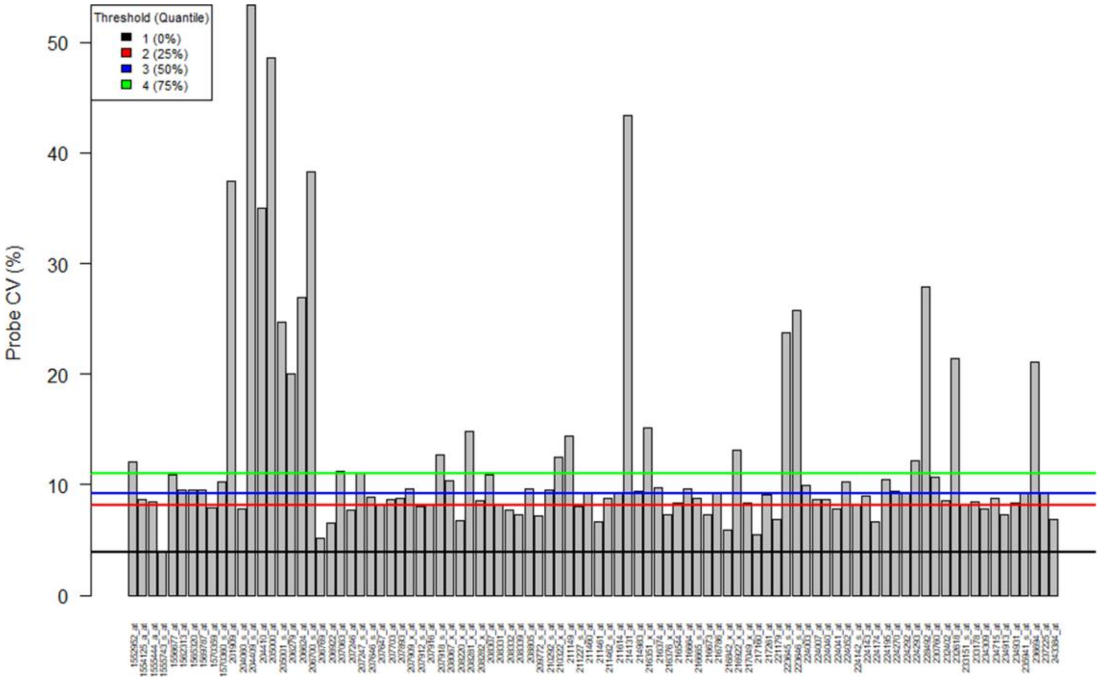

Supplementary Figure 1. The probe variation plot used to determine which genes to use in massiR R package [53]. A threshold of 4 was selected as it encompassed the genes with the highest variation and ignores genes with low variation that may be useful in classifying samples.

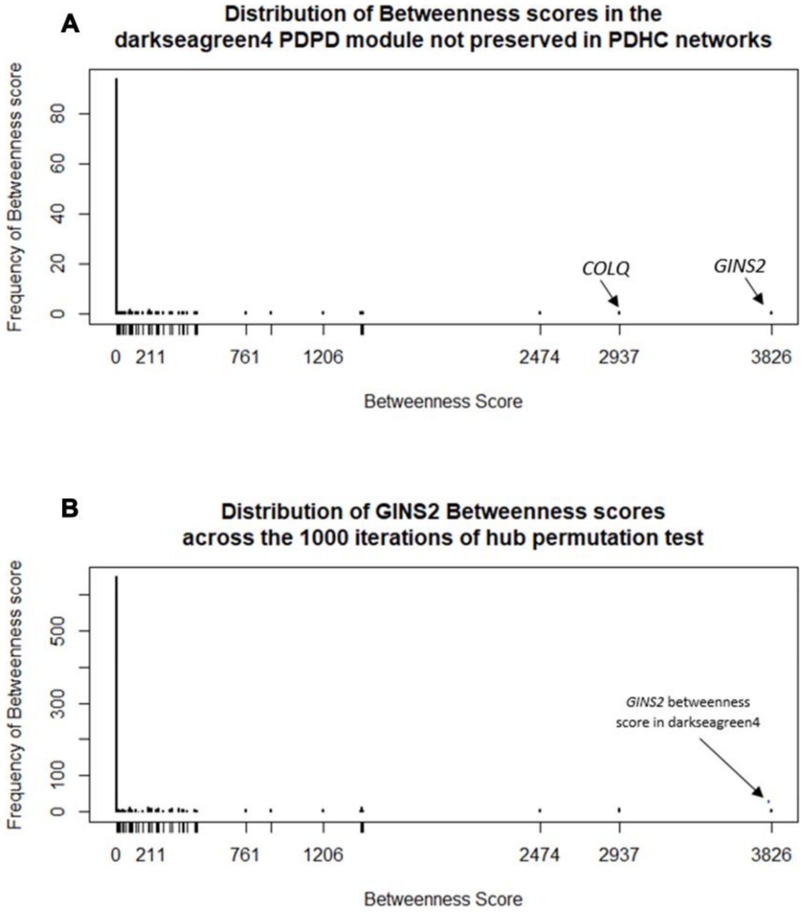

Supplementary Figure 2. (**A**) The distribution of betweenness scores for each gene in the darkseagreen4 module. Many genes have a betweenness score of 0 indicating they do not act as hubs in regard to betweenness in this module. After the hub permutation test, one gene was found to be significant (GINS2, Pvalue = 0.005). (**B**) The distribution of betweenness scores for GINS2 over the 1000 iterations of the hub permutation test. The betweenness score of GINS2 in the original darkseagreen4 module network is highlighted.
