# Supplementary material for: Genetic networks in Parkinson’s and Alzheimer’s disease
Source: Aging (Albany NY). 2020 Mar 23;12(6):5221–43. doi: 10.18632/aging.102943 (PMC7138567; doi:10.18632/aging.102943)
Supplement: Supplementary Tables 1, 3, 5-8 [file aging-12-102943-s003..pdf]

SUPPLEMENTARY TABLES

Supplementary Table 1. Information on number of samples, sex and age of samples in datasets.

GEO Dataset		No. sample	Sex (male/female)	Mean Age (±SD)
GSE99039	PD	204	97/107	NA
	HC	230	150/80	NA
	All	434	247/187	NA
GSE63060 +	AD	245	166/79	76.5 (± 6.6)
GSE63061	MCI	142	79/66	74.9(± 6.3)
	HC	182	110/72	73.6 (± 6.3)
	All	569	352/217	75.2 (±6.5)

Please browse Full Text version to see the data of Supplementary Table 2.

Supplementary Table 2. Excel table of differentially expressed genes found in the PD dataset.

Supplementary Table 3. Excel table of significant hubs found in non-preserved modules between PD and healthy controls.

Module	Gene	Hub detection method	Score	P-value
PD modules not preserved in HC				
Darkseagreen4	GINS2	Betweenness	3826	0.005
	S1PR5	Kleinberg's centrality; PageRank; MM	0.30751; 0.02637; 0.90234	0.006; 0.006; 0.007
	AGBL2	Closeness	10.00256	0.007
	NKG7	PageRank	0.02512	0.007
Navajowhite2	SNRNP70	PageRank; Kleinberg's centrality	0.02359; 0.27933	0.003; 0.007
	POPDC2	Closeness	18.03573	0.008
	CHKB	Kleinberg's centrality	0.28034	0.009
	MIR142	MM	0.85297	0.009
Salmon	TYSND1	PageRank; MM; Kleinberg's centrality	0.00978; 0.84787; 0.17499	0.002; 0.002; 0.008
	C17orf97	Closeness	4.4882	0.002
	HDAC6	Kleinberg's centrality; MM; PageRank	0.17867; 0.83636; 0.00958	0.003; 0.006; 0.007
	FAM114A1	Betweenness	12901	0.004
	ZNF804A	Betweenness; Closeness	12956; 4.27567	0.005; 0.007
	ABCD1	PageRank; MM	0.00904; 0.83955	0.006; 0.006
	ZNF526	PageRank	0.00908	0.006
	TMEM147-AS1	Betweenness	12566	0.008
	RENBP	PageRank	0.00823	0.009
HC modules not preserved in PD				
Purple	FAM110C	Closeness; Betweenness	0.72585; 33683	0.000; 0.002

TXLNGY	Betweenness	40661	0
PAK4	Kleinberg's centrality; Pagerank; MM	0.12262; 0.00467; 0.83401	0.001; 0.002; 0.003
GIGYF1	Kleinberg's centrality; PageRank; MM	0.12332; 0.00473; 0.85428	0.002; 0.002; 0.002
WDTC1	Kleinberg's centrality; MM; PageRank	0.11337; 0.82836; 0.00441	0.002; 0.004; 0.008
NEB	Closeness; Betweenness	0.70015; 21395	0.003; 0.004
SH3BGR	Closeness; Betweenness	0.63727; 19636	0.004; 0.005
FCGBP	Betweenness	16988	0.005
INO80B	PageRank; Kleinberg's centrality; MM	0.00417; 0.10391; 0.82766	0.005; 0.007; 0.007
ZNF582-AS1	Closeness; Betweenness	0.59408; 0.06978	0.006; 0.008
PLA2G4C	Betweenness	20491	0.007
TBC1D25	PageRank; MM	0.00401; 0.81547	0.007; 0.007
MFSD12	Kleinberg's centrality; PageRank; MM	0.10808; 0.00411; 0.80996	0.007; 0.009; 0.009
MCM2	Closeness	0.57973	0.008
SPATA6	Closeness	0.65087	0.009
RPS6KA4	MM	0.80597	0.009
FIZ1	MM	0.81009	0.009

Please browse Full Text version to see the data of Supplementary Table 4.

Supplementary Table 4. Excel table of significant hubs found in non-preserved modules between AD, MCI and healthy controls.

Supplementary Table 5. Excel file containing the significant TFs (Pvalue < 0.01) associated with each non-preserved module between PD and healthy control networks found using Enrichr (ENCODE and ChEA Consensus TFs from ChIP-X).

Module colour	Significant TFs	P-value	Gene overlap
PD modules not preserved in HC			
Darkseagreen4	FOXM1	4.004E-08	9/95
	E2F4	8.131E-08	21/710
Navajowhite2	RUNX1	0.008305	18/1294
Salmon	FOXM1	0.006578	6/95
HC modules not preserved in PD			
Purple	SIX5	0.0001626	55/1094
	ZBTB7A	0.0002814	94/2184
	SRF	0.0008434	20/299
	CREB1	0.001402	64/1444
	NFYB	0.004818	138/3715
	PBX3	0.007364	54/1269

Supplementary Table 6. Excel file containing the significant TFs (Pvalue < 0.01) associated with each non-preserved module between AD, MCI and healthy control networks found using Enrichr (ENCODE and ChEA Consensus TFs from ChIP-X).

Module colour	Significant TFs	P-value	Gene overlap
AD modules not preserved in HC and MCI			
Blue	SUZ12	3.36E-10	150/1684
	EZH2	0.0004579	26/237
MCI modules not preserved in AD and HC			
Sienna3	SUZ12	8.24E-10	115/1684
HC modules not preserved in AD and MCI			
Darkolivegreen	SUZ12	0.00392	68/1684
	REST	0.009205	20/383
Darkorange2	IRF3	0.000002884	24/663
	SP2	0.000006359	30/994
	NFYB	0.0000105	74/3715
	GABPA	0.00001689	48/2082
	BRCA1	0.0003388	61/3218
	CTCF	0.0003775	39/1790
	NFYA	0.0004409	46/2250
	PBX3	0.0005193	30/1269
	SIX5	0.00115	26/1094
	SMC3	0.003293	26/1181
	NR2C2	0.004466	11/350
	FOS	0.006121	16/637
	CREB1	0.007005	29/1444
Skyblue	RCOR1	0.002542	15/702
	BCLAF1	0.006338	16/851
HC modules not preserved in MCI			
Red	SUZ12	1.21E-09	107/1684
	EZH2	0.0001041	21/237

Supplementary Table 7. Excel file containing the SNPs associated with PD SNPs in bold are shared between PD and AD.

Chromosome	SNPs	Associated PD related miRNAs	Modules with SNP associated gene	Genes
1	rs12140193	hsa-miR-495	PD darkseagreen4	METTL13
2	rs1138729	hsa-miR-495	PD salmon	RRM2
	**rs12603**	hsa-miR-543	HC purple	EPB41L5
	rs2058703	hsa-miR-1283	HC purple; PD salmon	BCL11A
	rs4852735	hsa-miR-4271	PD navajowhite2	TEX261
	**rs707718**	hsa-miR-543	HC purple	CYP26B1
3	**rs1135750**	hsa-miR-147a	PD navajowhite2	IQCB1
	**rs11551405**	hsa-miR-203	HC purple	DCP1A
4	rs3805317	hsa-miR-203	HC purple	CLGN
5	rs2561659	hsa-miR-543	HC purple	AHRR
6	**rs12528857**	hsa-miR-203	HC purple; PD darkseagreen4; PD salmon; PD navajowhite2	TDRD6
	**rs1966**	hsa-miR-543	HC purple; PD darkseagreen4	PSORS1C1
7	**rs1044718**	hsa-miR-147a	HC purple; PD darkseagreen4; PD salmon	PARP12
8	**rs2929969**	hsa-miR-133b; hsa-miR-203	PD darkseagreen4	WISP1
9	rs7047770	hsa-miR-133b	HC purple; PD navajowhite2	C9orf139
	rs818055	hsa-miR-147a	HC purple; PD navajowhite2	LAMC3
10	rs1042192	hsa-miR-376b	HC purple	CYP2C18
11	**rs10832733**	hsa-miR-543	HC purple	PIK3C2A
	rs2512676	hsa-miR-147a	PD darkseagreen4; PD salmon	DLG2
	rs7126647	hsa-miR-543	PD navajowhite2	MRGPRX2
	rs9444	hsa-miR-495	HC purple	RNF169
14	**rs1054195**	hsa-miR-543	PD navajowhite2	CLMN
16	rs1568391	hsa-miR-495	PD darkseagreen4	IRF8
17	**rs3744711**	hsa-miR-203	HC purple; PD salmon	DHX33
18	rs1790974	hsa-miR-203	HC purple	DOK6
	rs3745067	hsa-miR-4271	HC purple; PD darkseagreen4; PD salmon	ONECUT2
19	rs36621	hsa-miR-376b	PD navajowhite2	TSEN34
20	rs1060347	hsa-miR-134	HC purple	PCMTD2
22	rs712979	hsa-miR-203	HC purple	C22orf39

Supplementary Table 8. Excel file containing the SNPs associated with AD SNPs in bold are shared between AD and PD.

Chromosome	SNPs	Associated PD related miRNAs	Modules with SNP associated gene	Genes
1	rs6660019	hsa-miR-433	AD blue; HC darkolivegreen; MCI sienna3	SASS6
2	**rs12603**	hsa-miR-543	HC darkorange2	EPB41L5
	**rs707718**	hsa-miR-543	AD blue; HC darkolivegreen; HC red; MCI sienna3	CYP26B1
3	**rs1135750**	hsa-miR-147a	HC skyblue	IQCB1
	**rs11551405**	hsa-miR-203	AD blue; HC darkorange2; HC red	DCP1A
	rs340833	hsa-miR-433	HC skyblue	IL5RA
	rs6792607	hsa-miR-153	HC skyblue	EIF5A2
4	**rs3805317**	hsa-miR-203	AD blue; HC red; MCI sienna3	CLGN
	rs8336	hsa-miR-203	AD blue	SMARCAD1
6	rs10864	hsa-miR-433	AD blue; HC red; MCI sienna3	BCKDHB
	**rs12528857**	hsa-miR-203	AD blue; HC darkorange2; HC red; MCI sienna3	TDRD6
	**rs1966**	hsa-miR-543	AD blue; HC red; MCI sienna3	PSORS1C1
	rs4709266	hsa-miR-433	AD blue; HC red; MCI sienna3	TAGAP
7	**rs1044718**	hsa-miR-147a	HC red	PARP12
8	rs1042992	hsa-miR-495	HC darkorange2	BNIP3L
	**rs2929969**	hsa-miR-133b; hsa-miR-203	AD blue	WISP1
	rs732338	hsa-miR-134	AD blue; HC red; MCI sienna3	LZTS1
10	rs7071789	hsa-miR-495	HC darkolivegreen	TRUB1
11	**rs10832733**	hsa-miR-543	HC darkorange2	PIK3C2A
14	**rs1054195**	hsa-miR-543	AD blue; MCI sienna3	CLMN
16	rs7294	hsa-miR-147a	HC darkolivegreen	VKORC1
17	**rs3744711**	hsa-miR-203	HC darkorange2; HC skyblue	DHX33
18	rs1046699	hsa-miR-433	AD blue; HC red; MCI sienna3	C18orf54
	rs608823	hsa-miR-433	AD blue; HC red; MCI sienna3	ONECUT2
21	rs243609	hsa-miR-543	AD blue; HC red; MCI sienna3	C21orf91
22	rs137124	hsa-miR-134	AD blue	CYB5R3
	rs17032	hsa-miR-495	HC darkolivegreen	SUN2
